# Relationship between time-weighted average glucose and mortality in critically ill patients: a retrospective analysis of the MIMIC-IV database

**DOI:** 10.1038/s41598-024-55504-9

**Published:** 2024-02-27

**Authors:** Mengwen Feng, Jing Zhou

**Affiliations:** 1 Department of Critical Care Medicine, The First Affiliated Hospital of Nanjing Medical University, Nanjing, 210029 China; 2 Department of Geriatric Intensive Care Medicine, The First Affiliated Hospital of Nanjing Medical University, Nanjing, 210029 China

**Keywords:** Critical care, Glycemia control, Mortality, Restricted cubic splines regression, MIMIC-IV, Diseases, Endocrinology, Medical research

## Abstract

Blood glucose management in intensive care units (ICU) remains a controversial topic. We assessed the association between time-weighted average glucose (TWAG) levels and ICU mortality in critically ill patients in a real-world study. This retrospective study included critically ill patients from the Medical Information Mart for Intensive Care IV database. Glycemic distance is the difference between TWAG in the ICU and preadmission usual glycemia assessed with glycated hemoglobin at ICU admission. The TWAG and glycemic distance were divided into 4 groups and 3 groups, and their associations with ICU mortality risk were evaluated using multivariate logistic regression. Restricted cubic splines were used to explore the non-linear relationship. A total of 4737 adult patients were included. After adjusting for covariates, compared with TWAG ≤ 110 mg/dL, the odds ratios (ORs) of the TWAG > 110 mg/dL groups were 1.62 (95% CI 0.97–2.84, *p* = 0.075), 3.41 (95% CI 1.97–6.15, *p* < 0.05), and 6.62 (95% CI 3.6–12.6, *p* < 0.05). Compared with glycemic distance at − 15.1–20.1 mg/dL, the ORs of lower or higher groups were 0.78 (95% CI 0.50–1.21, *p* = 0.3) and 2.84 (95% CI 2.12–3.82, *p* < 0.05). The effect of hyperglycemia on ICU mortality was more pronounced in non-diabetic and non-septic patients. TWAG showed a U-shaped relationship with ICU mortality risk, and the mortality risk was minimal at 111 mg/dL. Maintaining glycemic distance ≤ 20.1 mg/dL may be beneficial. In different subgroups, the impact of hyperglycemia varied.

## Introduction

Glycometabolism disorder is widespread in critically ill patients and mainly manifests as hyperglycemia^1–3^, which is associated with poor clinical outcomes^3–7^. Many studies have been conducted on hyperglycemia over the past 20 years, but the optimal glycemic target for critically ill patients remains controversial^8^. Single-center trials conducted by Van den Berghe et al. showed that patients who stayed in the intensive care unit (ICU) for three or more days, in-hospital mortality significantly reduced among those maintaining glycemia at 80–110 mg/dL^3^. However, the multicenter NICE-SUGAR study including 6104 critically ill patients found nearly opposite results in that maintaining glycemia below 180 mg/dL compared with intensive glycemic control (81–108 mg/dL) reduced 90-day mortality in critically ill patients (24.9% vs. 27.5%, *p* = 0.02)^7^. Another study suggested that mild glycemic control (120–144 mg/dL) can reduce negative nitrogen balance and thus benefit patients^9^. However, glycemia below 180 mg/dL has gradually become considered the conventional glycemia control target in critically ill patients^10–13^. The American Diabetes Association Standards of Medical Care in Diabetes Guideline^14^ and the 2021 Surviving Sepsis Campaign (SSC) Guidelines^15^ recommend maintaining glycemia within the range of 144–180 mg/dL in critically ill patients. Some studies have suggested that the range of glycemia associated with a lower mortality rate in patients with diabetes is higher than that in non-diabetic patients^16–18^. Intensive glycemic control can reduce the risk of in-hospital mortality among patients undergoing coronary artery bypass surgery^19^ as well as the rate of surgical site infection^20^. For patients with sepsis, different or even opposite results between hyperglycemia and mortality have been observed^12,21–23^. Preadmission glycemia and other disease states likely also play a role, and there is likely to be a complex non-linear relationship between hyperglycemia and prognosis in critically ill patients.

Several measures have been used in the literature to report blood glucose data and evaluate glycemic control. Unfortunately, a consistent method of describing glycemic control has not been used for this population. Using a time weighted average glucose (TWAG) eliminates the bias created by unequal time measurements and repeated testing around the same time, as time is factored into the calculation^24^. Based on the complexity and heterogeneity of critically ill patients, some scholars have proposed individualized glycemic targets in critically ill patients^25^. However, no further explanation has been given on how to individualize glycemic control strategies. A retrospective cohort study was performed to assess the association between glycemic control levels during ICU stay and ICU mortality, and to explore individualized glycemic targets related to usual glycemia.

## Results

### General information

**Figure 1:**
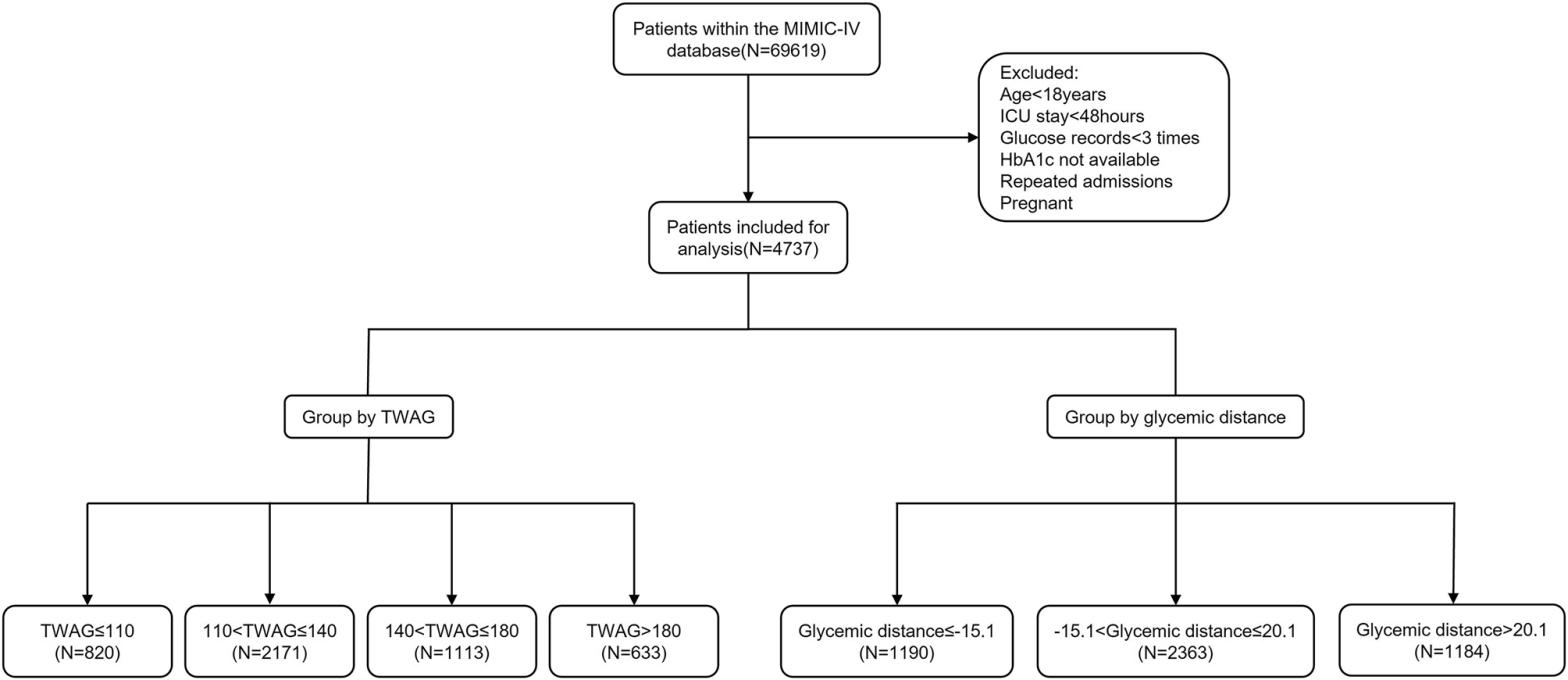
Flow chart of the study.

A total of 4737 patients were included in the analysis (Fig. 1). Demographic and clinical characteristics of the study participants are presented in Table 1. The median age of all patients was 69.6 years old, and 2853 (60.2%) patients were male. The incidence of diabetes was 44.1% (2090/4737), vasopressors was 52.4% (2484/4737), sepsis was 55.4% (2625/4737), hypoglycemia was 11% (522/4737), MV was 66.6% (3157/4737), and RRT was 6.5% (308/4737). The overall median TWAG and glycemic distance were 130 mg/dL and 3.2 mg/dL, respectively. The ICU and in-hospital mortality rates were 6.4% and 9.4%, respectively, and the median ICU and hospital stay were 3.9 days and 9.8 days, respectively. The TWAG of patients who died during ICU stay was significantly higher than that of survivors (129 [114, 154] vs. 145 [131, 178], *p* < 0.001). The survivor group also showed a significantly lower glycemic distance (2.3 [-15.7, 18.8] vs 23.6 [3.33, 43.4], *p* < 0.001) (Supplementary Table S1).

**Table 1:** Baseline demographic and clinical characteristics between time-weighted average glucose and glycemic distance groups.

Features	ALL	TWAG (mg/dL)				*p*-value	Glycemic distance (mg/dL)			*p*-value
		≤ 110	110–140	140–180	> 180		≤ − 15.1	− 15.1–20.1	> 20.1	
	N = 4737	N = 820	N = 2171	N = 1113	N = 633		N = 1190	N = 2363	N = 1184	
Demographic features										
Age, years	69.6 (59.1, 78.6)	70.0 (56.5, 80.9)	70.8 (61.1, 79.4)	68.7 (58.7, 77.7)	66.2 (55.3, 75.0)	< 0.001	66.8 (56.3, 76.9)	71.1 (60.3, 80.2)	69.3 (59.8, 77.2)	< 0.001
Male, no (%)	2853 (60.2)	462 (56.3)	1334 (61.4)	684 (61.5)	373 (58.9)	0.053	709 (59.6)	1426 (60.3)	718 (60.6)	0.858
BMI, kg/m^2^	28.0 (24.1, 32.9)	26.3 (22.9, 30.6)	27.8 (24.1, 32.2)	29.2 (24.8, 34.4)	29.8 (25.2, 35.2)	< 0.001	28.7 (24.3, 34.2)	27.8 (24.2, 32.5)	27.7 (23.9, 32.5)	< 0.001
Race, no (%)						< 0.001				< 0.001
White	2914 (61.5)	530 (64.6)	1409 (64.9)	639 (57.4)	336 (53.1)		673 (56.6)	1561 (66.1)	680 (57.4)	
Black	395 (8.34)	61 (7.44)	141 (6.49)	108 (9.7)	85 (13.4)		138 (11.6)	154 (6.52)	103 (8.7)	
Hispanic	169 (3.57)	22 (2.68)	59 (2.72)	51 (4.58)	37 (5.85)		66 (5.55)	62 (2.62)	41 (3.46)	
Asian	95 (2.0)	11 (1.34)	38 (1.75)	27 (2.43)	19 (3.0)		28 (2.35)	34 (1.44)	33 (2.79)	
Others	1164 (24.6)	196 (23.9)	524 (24.1)	288 (25.9)	156 (24.6)		285 (23.9)	552 (23.4)	327 (27.6)	
Vital signs										
Heart rate, bpm	81.9 (73.6, 91.8)	79.0 (70.1, 89.6)	81.1 (74.1, 89.6)	83.5 (74.6, 93.5)	87.8 (76.3, 97.5)	< 0.001	82.8 (74.4, 92.9)	80.9 (73.2, 89.8)	83.2 (74.4, 94.5)	< 0.001
MAP, mmHg	77.4 (71.6, 86.4)	79.8 (72.7, 90.2)	76.1 (71.0, 84.0)	77.7 (71.7, 86.0)	80.2 (73.1, 89.3)	< 0.001	76.6 (71.1, 85.7)	77.4 (71.4, 86.4)	78.1 (72.4, 87.0)	0.006
Respiratory rate, bpm	18.5 (16.7, 20.7)	18.1 (16.3, 20.0)	18.2 (16.6, 20.3)	18.9 (16.9, 21.4)	19.4 (17.6, 22.0)	< 0.001	18.2 (16.4, 20.2)	18.3 (16.6, 20.5)	19.0 (17.1, 21.6)	< 0.001
Temperature, ℃	36.8 (36.6, 37.1)	36.8 (36.6, 37.1)	36.8 (36.5, 37.1)	36.9 (36.6, 37.2)	36.9 (36.7, 37.3)	< 0.001	36.8 (36.6, 37.1)	36.8 (36.6, 37.1)	36.9 (36.6, 37.2)	< 0.001
SpO2, %	97.5 (96.1, 98.7)	97.4 (96.1, 98.5)	97.6 (96.3, 98.7)	97.6 (96.1, 98.7)	97.1 (95.6, 98.5)	< 0.001	97.6 (96.3, 98.7)	97.5 (96.1, 98.6)	97.5 (96.0, 98.8)	0.301
Severe of illness										
CCI	6 (4, 8)	6 (4, 8)	6 (4, 7)	6 (5, 8)	7 (5, 9)	< 0.001	6 (5, 8)	6 (4, 7)	6 (5, 8)	< 0.001
APS III	43 (32, 59)	38 (29, 51)	41 (31, 56)	46 (35, 65)	50 (38, 66)	< 0.001	43 (33, 56)	41 (30, 55)	48 (36, 68)	< 0.001
SOFA	5 (3, 8)	4 (2, 6)	5 (3, 8)	5 (3, 8)	5 (3, 8)	< 0.001	5 (3, 8)	5 (3, 7)	6 (4, 8)	< 0.001
Laboratory results										
WBC, × 10^9^/L	13.6 (10.1, 18.1)	11.3 (8.5, 15.2)	14.1 (10.5, 18.6)	14.4 (10.7, 18.5)	14.1 (10.4, 18.5)	< 0.001	13.1 (9.72, 17.7)	13.5 (10.1, 17.9)	14.6 (10.6, 18.9)	< 0.001
Hemoglobin, g/L	10.1 (8.4, 12.1)	10.8 (8.7, 12.5)	9.7 (8.2, 11.5)	10.1 (8.5, 12.1)	11.0 (9.4, 12.6)	< 0.001	10.0 (8.5, 12.1)	10.1 (8.4, 12.0)	10.3 (8.4, 12.1)	0.55
Platelets, × 10^9^/L	166 (122, 224)	175 (130, 232)	155 (114, 209)	170 (126, 232)	187 (144, 240)	< 0.001	176 (130, 236)	163 (121, 217)	163 (117, 221)	< 0.001
Creatinine, mg/dL	1.1 (0.8, 1.5)	1.0 (0.8, 1.4)	1.0 (0.8, 1.4)	1.1 (0.9, 1.6)	1.3 (0.9, 1.9)	< 0.001	1.1 (0.8, 1.5)	1.0 (0.8, 1.4)	1.1 (0.8, 1.7)	< 0.001
Prothrombin time, s	14.4 (12.6, 16.9)	13.8 (12.2, 16.3)	15.0 (13.0, 17.2)	14.3 (12.6, 16.8)	13.4 (12.1, 15.8)	< 0.001	14.3 (12.5, 16.6)	14.5 (12.6, 17.0)	14.2 (12.6, 16.9)	0.058
Lactate, mg/dL	2.1 (1.4, 3.1)	1.7 (1.1, 2.5)	2.2 (1.5, 3.1)	2.2 (1.5, 3.1)	2.0 (1.4, 3.2)	< 0.001	2.1 (1.4, 2.9)	2.1 (1.4, 3.0)	2.1 (1.5, 3.3)	0.028
HbA1c, %	5.9 (5.5, 6.9)	5.6 (5.2, 5.9)	5.7 (5.4, 6.1)	6.6 (5.9, 7.8)	8.0 (6.8, 10.2)	< 0.001	7.7 (6.3, 9.9)	5.7 (5.4, 6.2)	5.7 (5.2, 6.4)	< 0.001
Diabetes, no (%)	2090 (44.1)	127 (15.5)	600 (27.6)	782 (70.3)	581 (91.8)	< 0.001	900 (75.6)	685 (29.0)	505 (42.7)	< 0.001
Sepsis, no (%)	2625 (55.4)	368 (44.9)	1215 (56.0)	660 (59.3)	382 (60.3)	< 0.001	600 (50.4)	1278 (54.1)	747 (63.1)	< 0.001
Hypoglycemia, no (%)	522 (11)	179 (21.8)	196 (9.03)	101 (9.07)	46 (7.27)	< 0.001	240 (20.2)	218 (9.23)	64 (5.41)	< 0.001
severe hypoglycemia, no (%)	25 (0.53)	9 (1.1)	10 (0.46)	4 (0.36)	2 (0.32)	0.14	11 (0.92)	11 (0.47)	3 (0.25)	0.066
Treatment										
MV, no (%)	3157 (66.6)	400 (48.8)	1625 (74.9)	776 (69.7)	356 (56.2)	< 0.001	752 (63.2)	1585 (67.1)	820 (69.3)	0.006
MV duration, h	7.7 (0.0, 27.4)	0.0 (0.0, 16.0)	9.2 (0.0, 25.1)	10.6 (0.0, 45.0)	5.8 (0.0, 41.0)	< 0.001	6.95 (0.0, 22.6)	7.0 (0.0, 23.0)	12.0 (0.0, 50.2)	< 0.001
Vasopressors, no (%)	2484 (52.4)	323 (39.4)	1349 (62.1)	574 (51.6)	238 (37.6)	< 0.001	607 (51.0)	1269 (53.7)	608 (51.4)	0.217
RRT, no (%)	308 (6.5)	57 (6.95)	116 (5.34)	82 (7.37)	53 (8.37)	0.018	75 (6.3)	125 (5.29)	108 (9.12)	< 0.001
Mean insulin dose, IU/day	9.1 (0.0, 32.2)	0.0 (0.0, 5.2)	8.7 (0.0, 25.6)	16.2 (2.0, 49.9)	28.4 (11.5, 72.9)	< 0.001	28.4 (1.8, 62.1)	5.0 (0.0, 22.5)	6.9 (0.5, 22.5)	< 0.001
Length of ICU stay, days	3.9 (2.7, 6.1)	3.7 (2.77, 5.1)	3.9 (2.7, 6.2)	3.9 (2.8, 7.1)	3.9 (2.7, 6.2)	0.001	3.5 (2.6, 5.4)	3.9 (2.8, 6.0)	4.2 (2.8, 7.5)	< 0.001
ICU mortality, no (%)	302 (6.38)	21 (2.6)	112 (5.2)	96 (8.6)	73 (11.5)	< 0.001	38 (3.2)	101 (4.3)	163 (13.8)	< 0.001
Length of hospital stay, days	9.8 (6.7, 15.0)	8.6 (5.8, 12.9)	9.9 (6.9, 14.8)	10.2 (6.9, 16.7)	9.7 (6.1, 16.1)	< 0.001	9.7 (6.8, 14.7)	9.5 (6.6, 14.1)	10.6 (6.8, 16.7)	0.001
Hospital mortality, no (%)	443 (9.35)	44 (5.4)	168 (7.7)	131 (11.8)	103 (16.3)	< 0.001	71 (6.0)	155 (6.6)	220 (18.6)	< 0.001

Continuous variables are presented as median with interquartile range (IQR) and were compared between groups using Kruskal–Wallis tests. Categorical variables are presented as numbers and percentage and were compared between groups using the chi-square. Statistical significance was set at *p* < 0.05.

*TWAG* time-weighted average glucose; *BMI* body mass index; *MAP* mean arterial pressure; *CCI* Charlson Comorbidity Index; *APS III* Acute Physiology Score III; *SOFA* Sequential Organ Failure Assessment; *WBC* white blood cell; *HbA1c* glycated hemoglobin; *MV* mechanical ventilation; *RRT* renal replacement therapy.

Patients with higher TWAG tended to have higher BMI, heart rate, APS III, creatinine, mean insulin dose, diabetes, and RRT. The SOFA score, WBC count, lactate, and sepsis in patients with TWAG ≤ 110 mg/dL were decreased than those in other groups, but hypoglycemia (21.8%) was increased. An upward trend was observed at higher glycemic distance levels for mean arterial pressure (MAP), respiratory rate, temperature, WBC, SOFA score, MV duration time, and sepsis.

### Association between TWAG, glycemic distance, and outcomes

The rates of ICU mortality in the four TWAG groups (≤ 110 mg/dL, 110–140 mg/dL, 140–180 mg/dL, and > 180 mg/dL) were 2.6%, 5.2%, 8.6%, and 11.5%, respectively (Table 1). Compared with TWAG ≤ 110 mg/dL, the odds ratios (ORs) for TWAG > 110 mg/dL were 1.62 (95% CI 0.97–2.84, *p* = 0.075), 3.41 (95% CI 1.97–6.15; *p* < 0.05), and 6.62 (95% CI 3.6–12.6; *p* < 0.05) (Table 2, Model 3). ICU mortality according to groups of glycemic distance ≤  − 15.1 mg/dL, − 15.1–20.1 mg/dL, and > 20.1 mg/dL, were 3.2%, 4.3%, and 13.8% (Table 1). Compared with the group − 15.1–20.1 mg/dL, the ORs for glycemic distance ≤  − 15.1 mg/dL and > 20.1 mg/dL were 0.78 (95% CI 0.50–1.21; *p* = 0.3) and 2.84 (95% CI 2.12–3.82; *p* < 0.05), respectively (Table 2, Model 3).

**Table 2:** Odds ratio of ICU mortality according to time-weighted average glucose and glycemic distance groups.

	N	Event	OR (95% CI)	*p*	OR (95% CI)	*p*	OR (95% CI)	*p*
			Model 1		Model 2		Model 3	
TWAG (mg/dL)								
≤ 110	820	21	Ref		Ref		Ref	
110–140	2171	112	2.07 (1.32, 3.41)	0.003	1.71 (1.06, 2.89)	0.035	1.62 (0.97, 2.84)	0.075
140–180	1113	96	3.59 (2.27, 5.96)	< 0.001	2.39 (1.46, 4.07)	< 0.001	3.41 (1.97, 6.15)	< 0.001
> 180	633	73	4.96 (3.07, 8.35)	< 0.001	3.02 (1.80, 5.25)	< 0.001	6.62 (3.60, 12.6)	< 0.001
Glycemic distance (mg/dL)								
≤ − 15.1	1190	38	0.74 (0.50, 1.07)	0.12	0.61 (0.40, 0.90)	0.016	0.78 (0.50, 1.21)	0.3
− 15.1–20.1	2363	101	Ref		Ref		Ref	
> 20.1	1184	163	3.58 (2.77, 4.64)	< 0.001	2.70 (2.04, 3.58)	< 0.001	2.84 (2.12, 3.82)	< 0.001

Model 1: unadjusted model. Model 2: adjusted for age, gender, CCI, SOFA, APS III scores. Model 3: adjusted for age, gender, CCI, SOFA, APS III scores, diabetes, sepsis, hypoglycemia, MV, RRT, vasopressors, insulin.

**Figure 2:**
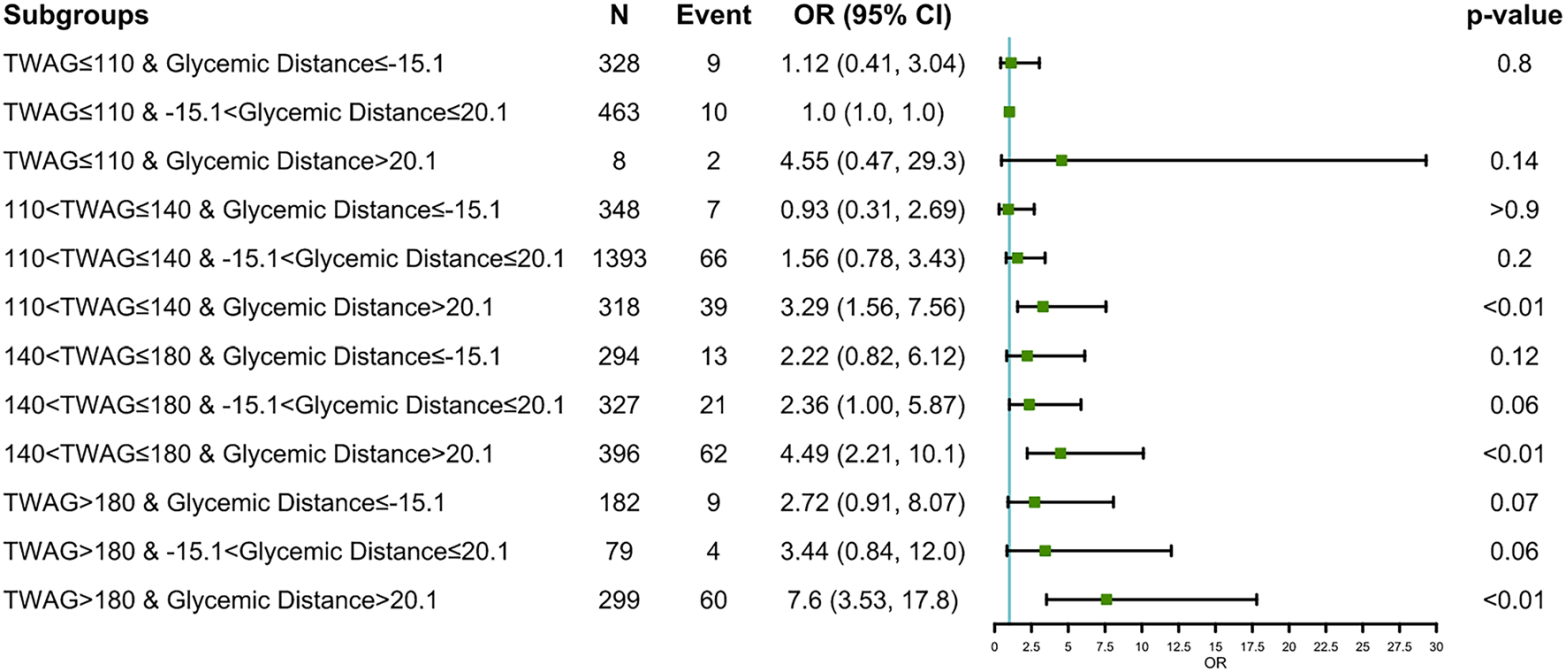
Forest plot depicting ICU mortality risk in critically ill patients. Adjustment factors are the same as those in Model 3. Grouped by different levels of time-weighted average glucose (TWAG) in combination with glycemic distance, and TWAG < 110 mg/dL plus − 15.1 < glycemic distance ≤ 20.1 mg/dL group was set as control group.

Furthermore, we analyzed the relationship between TWAG in combination with various levels of glycemic distance and ICU mortality risk (Fig. 2). Compared with TWAG < 110 mg/dL plus − 15.1 < glycemic distance ≤ 20.1 mg/dL, TWAG 110–140 mg/dL plus glycemic distance ≤  − 15.1 mg/dL showed a lower risk of ICU mortality, while the difference did not reach statistical significance. The ORs showed an increasing trend with increased TWAG and glycemic distance; however, the risk of ICU mortality was significantly increased only with TWAG > 110 mg/dL at the same time as glycemic distance > 20.1 mg/dL (*p* < 0.01).

### Subgroup analysis

Subgroup analyses indicated that in almost all subgroups, the risk of ICU mortality increased with elevated glycemia (Tables 3, 4). The effect of TWAG on ICU mortality was more pronounced in older, non-diabetic, and non-septic patients. Additionally, a significant interaction effect was observed between diabetes (*p* = 0.002), sepsis (*p* = 0.047), hypoglycemia (*p* = 0.021) and TWAG levels. We observed that patients who experienced at least one hypoglycemia event had higher ICU mortality rates than the corresponding subgroup at the same blood glucose level. The effect of glycemic distance on ICU mortality was more pronounced in non-diabetic patients. Additionally, a significant interaction effect was observed between diabetes (*p* < 0.001), sepsis (*p* = 0.021), hypoglycemia (*p* = 0.046) and glycemic distance levels.

**Table 3:** Odds ratio of ICU mortality according to time-weighted average glucose among subgroups.

Subgroups	N (event)	OR (95% CI)	*p*	Subgroups	N (event)	OR (95% CI)	*p*	*P* for interaction
Age < 65				Age ≥ 65				0.370
TWAG ≤ 110	318 (9)	1		TWAG ≤ 110	502 (12)	1		
110 < TWAG ≤ 140	735 (29)	0.80 (0.34, 2.04)	0.6	110 < TWAG ≤ 140	1436 (83)	2.48 (1.29, 5.21)	0.01	
140 < TWAG ≤ 180	448 (34)	2.72 (1.12, 7.17)	0.033	140 < TWAG ≤ 180	665 (62)	4.27 (2.11, 9.31)	< 0.001	
TWAG > 180	288 (26)	4.68 (1.73, 13.6)	0.003	TWAG > 180	345 (47)	8.54 (3.95, 19.7)	< 0.001	
Male				Female				0.980
TWAG ≤ 110	462 (12)	1		TWAG ≤ 110	358 (9)	1		
110 < TWAG ≤ 140	1334 (57)	1.30 (0.65, 2.80)	0.5	110 < TWAG ≤ 140	837 (55)	2.27 (1.07, 5.36)	0.044	
140 < TWAG ≤ 180	684 (60)	3.79 (1.83, 8.41)	< 0.001	140 < TWAG ≤ 180	429 (36)	3.15 (1.36, 7.92)	0.01	
TWAG > 180	373 (47)	7.63 (3.41, 18.2)	< 0.001	TWAG > 180	260 (26)	5.84 (2.30, 15.9)	< 0.001	
Diabetes				No Diabetes				0.002
TWAG ≤ 110	127 (3)	1		TWAG ≤ 110	693 (18)	1		
110 < TWAG ≤ 140	600 (27)	1.13 (0.36, 5.09)	0.8	110 < TWAG ≤ 140	1571 (85)	2.04 (1.13, 3.87)	0.022	
140 < TWAG ≤ 180	782 (44)	1.60 (0.52, 7.08)	0.5	140 < TWAG ≤ 180	331 (52)	5.74 (2.98, 11.6)	< 0.001	
TWAG > 180	581 (58)	3.18 (1.02, 14.2)	0.075	TWAG > 180	52 (15)	11.4 (4.55, 28.7)	< 0.001	
Sepsis				No Sepsis				0.047
TWAG ≤ 110	368 (19)	1		TWAG ≤ 110	452 (2)	1		
110 < TWAG ≤ 140	1215 (97)	1.34 (0.76, 2.45)	0.3	110 < TWAG ≤ 140	956 (15)	3.54 (0.88, 24.2)	0.12	
140 < TWAG ≤ 180	660 (81)	2.57 (1.41, 4.88)	0.003	140 < TWAG ≤ 180	453 (15)	11.6 (2.70, 81.8)	0.003	
TWAG > 180	382 (62)	4.89 (2.52, 9.87)	< 0.001	TWAG > 180	251 (11)	29.0 (5.77, 227)	< 0.001	
Hypoglycemia				No Hypoglycemia				0.021
TWAG ≤ 110	179 (11)	1		TWAG ≤ 110	641 (10)	1		
110 < TWAG ≤ 140	196 (25)	1.99 (0.83, 5.03)	0.13	110 < TWAG ≤ 140	1975 (87)	1.66 (0.86, 3.53)	0.2	
140 < TWAG ≤ 180	101 (15)	4.21 (1.36, 13.7)	0.014	140 < TWAG ≤ 180	1012 (81)	3.55 (1.80, 7.70)	< 0.001	
TWAG > 180	46 (7)	6.33 (1.60, 25.2)	0.008	TWAG > 180	587 (66)	6.93 (3.33, 15.7)	< 0.001	

Adjusted for model 3.

**Table 4:** Odds ratio of ICU mortality according to glycemic distance among subgroups.

Subgroups	N (event)	OR (95% CI)	*p*	Subgroups	N (event)	OR (95% CI)	*p*	*P* for interaction
Age < 65				Age ≥ 65				0.944
Glycemic distance ≤ − 15.1	531 (18)	1.76 (0.82, 3.75)	0.14	Glycemic distance ≤ − 15.1	659 (20)	0.47 (0.26, 0.83)	0.012	
− 15.1 < glycemic distance ≤ 20.1	815 (24)	1		− 15.1 < glycemic distance ≤ 20.1	1548 (77)	1		
Glycemic distance > 20.1	443 (56)	3.73 (2.17, 6.61)	< 0.001	Glycemic distance > 20.1	741 (107)	2.55 (1.79, 3.66)	< 0.001	
Male				Female				0.241
Glycemic distance ≤ − 15.1	709 (28)	1.24 (0.70, 2.15)	0.5	Glycemic distance ≤ − 15.1	481 (10)	0.36 (0.15, 0.78)	0.014	
− 15.1 < Glycemic distance ≤ 20.1	1426 (51)	1		− 15.1 < Glycemic distance ≤ 20.1	937 (50)	1		
Glycemic distance > 20.1	718 (97)	3.37 (2.26, 5.08)	< 0.001	Glycemic distance > 20.1	466 (66)	2.51 (1.61, 3.93)	< 0.001	
Diabetes				No Diabetes				< 0.001
Glycemic distance ≤ − 15.1	900 (30)	0.64 (0.37, 1.11)	0.12	Glycemic distance ≤ − 15.1	290 (8)	0.77 (0.30, 1.70)	0.5	
− 15.1 < glycemic distance ≤ 20.1	685 (41)	1		− 15.1 < glycemic distance ≤ 20.1	1678 (60)	1		
Glycemic distance > 20.1	505 (61)	1.59 (1.0, 2.56)	0.053	Glycemic distance > 20.1	679 (102)	4.17 (2.84, 6.19)	< 0.001	
Sepsis				No Sepsis				0.021
Glycemic distance ≤ − 15.1	600 (35)	0.85 (0.52, 1.35)	0.5	Glycemic distance ≤ − 15.1	590 (3)	0.46 (0.1, 1.54)	0.3	
− 15.1 < glycemic distance ≤ 20.1	1278 (87)	1		− 15.1 < glycemic distance ≤ 20.1	1085 (14)	1		
Glycemic distance > 20.1	747 (137)	2.62 (1.9, 3.65)	< 0.001	Glycemic distance > 20.1	437 (26)	3.86 (1.88, 8.19)	< 0.001	
Hypoglycemia				No Hypoglycemia				0.046
Glycemic distance ≤ − 15.1	240 (14)	0.87 (0.37, 1.99)	0.7	Glycemic distance ≤ − 15.1	950 (24)	0.77 (0.45, 1.29)	0.3	
− 15.1 < glycemic distance ≤ 20.1	218 (27)	1		− 15.1 < glycemic distance ≤ 20.1	2145 (74)	1		
Glycemic distance > 20.1	64 (17)	2.44 (1.03, 5.72)	0.04	Glycemic distance > 20.1	1120 (146)	3.01 (2.18, 4.17)	< 0.001	

Adjusted for model 3.

### Curve fitting

**Figure 3:**
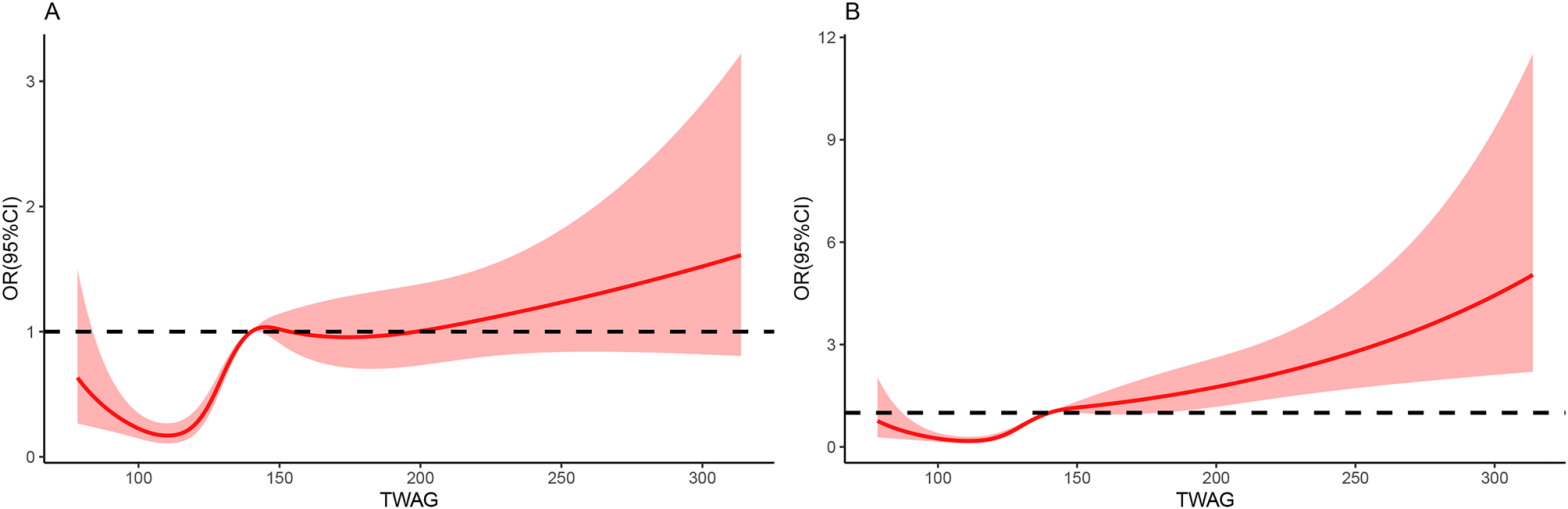
Odds ratios of ICU mortality as a function of time-weighted average glucose. Solid red lines are odds ratios, with light red regions showing 95% confidence intervals derived from restricted cubic spline regressions with five knots. The reference was set at 140 mg/dL. TWAG (**A**) was modeled as continuous variable and fitted in unadjusted model using restricted cubic splines. TWAG (**B**) was modeled as continuous variable and adjusted by factors are the same as those in Model 3.

The RCS results after multivariable adjustment flexibly modeled and visualized the relationship between TWAG on a continuous scale and the risk of ICU mortality (Fig. 3). The concentration of TWAG associated with the lowest risk of ICU mortality was 110 mg/dL in the univariate analysis (Fig. 3A). After adjusting for covariates, the risk reached a minimum when the concentration of TWAG was around 111 mg/dL; the value of OR increased when TWAG was lower or higher than this concentration (Fig. 3B).

## Discussion

In our study, TWAG showed a U-shaped relationship with ICU mortality risk. The ICU mortality risk with glycemic distance > 20.1 mg/dL significantly increased (*p* < 0.001). The effect of hyperglycemia on ICU mortality risk was more pronounced in patients without diabetes or sepsis.

The debate regarding the effect of hyperglycemia in critically ill patients has been ongoing for decades, but there is no consensus on the optimal glycemic target and how to perform individualized glycemic control remains unknown. The large multicenter RCT NICE-SUGAR demonstrated that the 90-day mortality in patients with blood glucose below 180 mg/dL was lower than that in patients maintaining blood glucose at 81–108 mg/dL(24.9% vs. 27.5%, *p* = 0.02)^7^. The American Diabetes Association Standards of Medical Care in Diabetes Guideline also recommends that glycemia should be maintained at 144–180 mg/dL in critically ill patients^14^. Previous studies have confirmed that the occurrence of hypoglycemia could increase patients’ mortality risk^26–28^. Tight systemic glucose control in patients with severe brain injury could reduce cerebral extracellular glucose availability and increase the prevalence of brain energy crisis, which in turn correlates with increased mortality^29^. Hyperglycemia is considered an adaptive response to stress, and mild to moderate hyperglycemia can benefit the body^23,30,31^. The reduction in the potential benefit of stress-related hyperglycemia and the high rate of hypoglycemia could explain the higher mortality rate in intensive glycemia control group.

In our study, the ICU mortality risk of hypoglycemia subgroups was higher than that of patients with similar glycemia levels but who did not experience hypoglycemia, which similar with previous studies. The incidence of hypoglycemia with TWAG ≤ 110 mg/dL and glycemic distance ≤  − 15.1 mg/dL (21.8%, 20.2%, respectively) was significantly higher than that in the other groups (*p* < 0.001), but mortality did not correspondingly increase. It is worth mentioning that the incidence of severe hypoglycemia with TWAG ≤ 110 mg/dL in this study was 1.1%, whereas in the studies by Bohé et al.^32^, Leuven^3,33^, and NICE-SUGAR^7^, the incidence in the intensive glycemia control groups was 3.9%, 5.1% and 6.8%, respectively. Strong glycemic management skills may be an important reason for the good prognosis with TWAG ≤ 110 mg/dL in this study.

Previous studies have calculated mean blood glucose without considering the time interval of blood glucose measurement, and have set fixed glycemia control targets for both conventional and intensive glycemia control groups^3,7,9–11^. Bohé et al. randomized critically ill patients to either an individualized glycemia control group with a glycemic target of usual glycemia + 15 mg/dL, or to a conventional glycemia control group^32^. The results showed no obvious differences in the risk of 28- and 90-day mortality between the two groups. We considered glycemic control during ICU hospitalization, in combination with usual glycemia levels. And we found that the risk of ICU mortality was significantly increased only in patients with TWAG > 110 mg/dL and glycemic distance > 20.1 mg/dL.

Patients with diabetes have a greater probability of developing chronic hyperglycemia, which can lead to mitochondrial damage and endothelial dysfunction. However, most published articles support that increased glycemia does not seem to have an obvious adverse impact on the risk of ICU mortality in patients with diabetes^16–18,23,34–38^. Similarly, the interaction test proved that hyperglycemia affects non-diabetic patients more than diabetic patients. Patients with diabetes can tolerate higher glycemia levels than non-diabetic patients, which may be partly due to long-term tolerance to high blood glucose, insulin resistance, and oxidative stress status^2^. One study found that for patients with diabetes, the incidence of relative hypoglycemia (glycemic distance > 30% below baseline) in the liberal blood glucose control group was higher than that in the intensive control group, but the ICU stay, ICU mortality, hospital stay, and in-hospital mortality did not show a significant difference^39^. We did not find different glycemic distance levels during the ICU stay increased the ICU mortality risk for diabetic patients.

Sepsis is a life-threatening organ dysfunction caused by dysregulation of the body's response to infection^40^. The influence of hyperglycemia in non-sepsis patients was much more obvious than that in sepsis patients, with OR increasing higher and faster. Patients with sepsis are prone to hypoglycemia, which is associated with an increased risk of mortality^26^. Patients with sepsis may benefit from a reduced incidence of hypoglycemia when blood glucose is elevated. The 2021 SSC guidelines recommend that glucocorticoids can be used in patients with septic shock^15^. Glucocorticoids can affect metabolism^41^. The use of glucocorticoids in sepsis patients may improves prognosis while causing an increase in blood glucose. This may also be part of the reason why sepsis patients tolerate higher blood glucose.

Our study also has some limitations. First, this was a single-center retrospective cohort study; prospective randomized controlled trials are needed to validate our findings. Second, this study failed to incorporate diagnosis into the analysis, and the metabolic impact of different disease pathological processes is different. In addition, TWAG reflects the average level of glycemia during the ICU stay but cannot reflect the glycemic change trend with a change in illness and insulin resistance. Furthermore, glycemia measurement methods influence the accuracy of the results, and using arterial (or venous) blood samples with classical laboratory devices or blood gas/glucose analyzers are better than bedside glucometers. However, these factors were not included in this analysis. More research are required to explore personalized glycemic control ranges for critically ill patients.

## Conclusions

TWAG showed a U-shaped relationship with ICU mortality risk, and the mortality risk was minimal at 111 mg/dL. Maintaining glycemic distance ≤ 20.1 mg/dL may be beneficial.

## Materials and methods

### Data sources and participants

The Massachusetts Institute of Technology established the Medical Information Mart for Intensive Care IV database (MIMIC-IV; version 2.2), which is a publicly and freely available database that contains critical care data of 73,181 patients of Beth Israel Deaconess Medical Center from 2008 to 2019^42^. Most high-quality data in MIMIC-IV are from the customized hospital electronic medical record system and the clinical information system of the ICU. Users can screen for demographic characteristics, vital signs, laboratory test results, and drug data. After passing the “Protecting Human Research Participants” exam on the National Institutes of Health website, one author (Mengwen Feng) was approved to extract data from the database (Record ID: 10,764,428). All research was performed in accordance with relevant guidelines and regulations.

All adult patients (age ≥ 18 years) with available glycated hemoglobin (HbA1c) records at ICU admission were screened for analysis. We excluded patients who remained in the ICU for less than 48 h and those with less than three blood glucose measurements during ICU stay to avoid inaccurate evaluation of glycemic fluctuations. Pregnant patients were excluded from the analysis. For patients with records of multiple admissions or ICU stays, we only included data of the first ICU stay.

### Data collection

Patients’ variables were extracted using Postgre SQL tools, including (1) demographic features (age, sex, race), body mass index (BMI); (2) vital signs, laboratory data, Sequential Organ Failure Assessment (SOFA) score, Acute Physiology Score III (APS III), Charlson Comorbidity Index (CCI) within the first 24 h after ICU admission; (3) anamnesis (diabetes), HbA1c records at ICU admission, and blood glucose records during the ICU stay; (4) use of insulin, mechanical ventilation (MV), renal replacement therapy (RRT), vasopressors, and incidence of sepsis during the ICU stay; (5) length of hospital stay and ICU stay, ICU mortality, and in-hospital mortality of all patients. Hypoglycemia was defined as glycemia < 72 mg/dL during ICU stay, and severe hypoglycemia was defined as glycemia < 40 mg/dL during ICU stay. Patients were considered to have diabetes if they had a medical history of diabetes and/or an HbA1c level of ≥ 6.5%. The diagnosis of sepsis was based on the criteria of the Third International Consensus Definitions for Sepsis and Septic Shock (Sepsis-3), which define sepsis as SOFA score ≧2 and the presence of infection or suspected infection^40^. Vasopressors included epinephrine, norepinephrine, dopamine, dobutamine and phenylephrine.

### Glucose measurement and glycemic distance definition

For each patient included in the analysis, we assessed preadmission glycemia using the patient’s HbA1c records:

$$ usual \;glycemia = 28.7 \times HbA1c - 46.7 $$

(in mg/dL, with HbA1c in %)^43^. To minimize the influence of variations in sampling intervals, the time-weighted average glucose (TWAG) was calculated for each patient^24^. The *i*th blood glucose measurement was recorded as Gi, the time interval between the *i*th and the *i* + 1th blood glucose measurement was recorded as ΔTi, and the last ΔTi was recorded as the time interval between the last Gi and the time of discharge or death:

$$ TWAG = {\Sigma }\left( {Gi \times \Delta Ti} \right) \div {\Sigma }\Delta Ti $$

(in mg/dL, with ΔTi in %). Then, we defined glycemic distance as the difference between TWAG and usual glycemia.

TWAG was stratified as follows: ≤ 110 mg/dL, 110–140 mg/dL, 140–180 mg/dL, and > 180 mg/dL. We grouped glycemic distance into three categories according to percentiles (low: ≤ 25th; mild: 25–75th; high: > 75th): ≤  − 15.1 mg/dL, − 15.1–20.1 mg/dL, and > 20.1 mg/dL. Here, we considered TWAG ≤ 110 mg/dL and − 15.1 < glycemic distance ≤ 20.1 mg/dL as reference values to which each category was compared.

### End points

The primary endpoint was ICU mortality. Secondary endpoints were in-hospital mortality, length of ICU stay, and length of hospital stay.

### Statistical analysis

Continuous variables are presented as mean ± standard deviation or median with interquartile range (IQR) and were compared between groups using one-way ANOVA or Kruskal–Wallis tests. Categorical variables are presented as numbers and percentage and were compared between groups using the chi-square or Fisher’s exact test. Missing values were imputed using a random forest function, and variables with > 20% missing values were deleted. Outliers, defined as values greater than the 99th percentile or lower than the 1st percentile, were winsorized.

Logistic regression was used to explore the association between TWAG, glycemic distance and ICU mortality in critically ill patients. The model was adjusted for potential confounders. Initially, we adjusted for age, sex, CCI, SOFA, and APS III scores (model 2). Subsequently, we adjusted for diabetes, sepsis, hypoglycemia, and related interventions, such as MV, RRT, vasopressors, and insulin (model 3).

In the subgroup analysis, we stratified the patients by age (≥ 65, < 65 years), sex (male, female), diabetes, sepsis, and hypoglycemia. The interaction of different levels of TWAG and glycemic distance with the above covariates for stratification of ICU mortality was examined by including two-factor interaction terms in the multivariate logistic regression model.

Given the hypothesis that the relationship between TWAG, glycemic distance, and risk of ICU mortality is non-linear, we further used a multivariate logistic model with restricted cubic splines (RCS) with five knots (10th, 25th, 50th, 75th, and 90th percentiles) for TWAG and glycemic distance. The reference level was set at 140 mg/dL. All statistical analyses were performed using R 4.2.2 (The R Project for Statistical Computing, Vienna Austria) software. Two-sided *p*-values < 0.05 were taken to indicate statistical significance.

### Ethical statements

The MIMIC-IV database was approved by the Massachusetts Institute of Technology (Cambridge, MA) and Beth Israel Deaconess Medical Center (Boston, MA), and consent was obtained for the collection of original data. Therefore, ethical approval and the need for informed consent were waived for this study.

### Supplementary Information

Supplementary Table S1.

## Data Availability

The data presented in this study are available on request from the corresponding author. The link of MIMIC IV dataset: https://physionet.org/content/mimiciv/2.2/.

## Abbreviations

ICU: Intensive care unit
TWAG: Time-weighted average glucose
MIMIC-IV: Medical Information Mart for Intensive Care IV
IQR: Interquartile range
RCS: Restricted cubic splines
SSC: Surviving Sepsis Campaign
HbA1c: Glycated hemoglobin
BMI: Body mass index
MAP: Mean arterial pressure
SOFA: Sequential Organ Failure Assessment
CCI: Charlson Comorbidity Index
APS III: Acute Physiology Score III
WBC: White blood cell
MV: Mechanical ventilation
RRT: Renal replacement therapy
MBG: Mean blood glucose
