# Supplementary material for: Relationship between time-weighted average glucose and mortality in critically ill patients: a retrospective analysis of the MIMIC-IV database
Source: Sci Rep. 2024 Feb 27;14:4721. doi: 10.1038/s41598-024-55504-9 (PMC10899565; doi:10.1038/s41598-024-55504-9)
Supplement: Supplementary file 1 — Supplementary Table S1. [file 41598_2024_55504_MOESM1_ESM.pdf]

Relationship between time-weighted average glucose and mortality in critically ill patients: a retrospective analysis of the MIMIC-IV database

Mengwen Feng [1], Jing Zhou [2,*]

Table S1: Comparison between survivors and non-survivors during the ICU stay.

**Features**	Total N=4737	Survivors N=4435	Non-survivors N=302	**p-value**
Demographic features				
Age, years	69.6 (59.1, 78.6)	69.4 (59.0, 78.5)	72.7 (60.1, 81.5)	0.011
Male, no (%)	2853 (60.2)	2677 (60.4)	176 (58.3)	
BMI, kg/m^2	28.0 (24.1, 32.9)	28.0 (24.1, 32.9)	27.2 (23.7, 33.4)	0.389
Race, no (%)				<0.001
White	2914 (61.5)	2778 (62.6)	136 (45.0)	
Black	395 (8.3)	369 (8.3)	26 (8.6)	
Hispanic	169 (3.6)	156 (3.5)	13 (4.3)	
Asian	95 (2.0)	88 (2.0)	7 (2.3)	
Others	1164 (24.6)	1044 (23.5)	120 (39.7)	
Vital signs				
Heart rate, bpm	81.9 (73.6, 91.8)	81.9 (73.8, 91.5)	82.2 (70.9, 96.5)	0.595
MAP, mmHg	77.4 (71.6, 86.4)	77.4 (71.6, 86.5)	78.0 (71.0, 85.7)	0.886
Respiratory rate, bpm	18.5 (16.7, 20.7)	18.3 (16.6, 20.6)	20.1 (18.0, 23.3)	<0.001
Temperature, °C	36.8 (36.6, 37.1)	36.8 (36.6, 37.1)	36.9 (36.5, 37.4)	0.014
SpO2, %	97.5 (96.1, 98.7)	97.5 (96.1, 98.6)	97.9 (96.4, 99.1)	0.005
Severe of illness				
CCI	6 (4, 8)	6 (4, 8)	7 (5, 9)	<0.001
APS III	43.0 (32.0, 59.0)	42.0 (31.0, 55.5)	76.0 (56.0, 96.8)	<0.001
SOFA	5 (3, 8)	5 (3, 7)	8 (5, 11)	<0.001
Laboratory results				
WBC, ×10^9/L	13.6 (10.1, 18.1)	13.5 (10.0, 17.9)	15.8 (11.8, 20.4)	<0.001
Hemoglobin, g/L	10.1 (8.4, 12.1)	10.1 (8.4, 12.0)	10.7 (8.4, 12.3)	0.115
Platelets, ×10^9/L	166 (122, 224)	166 (122, 224)	172 (120, 224)	0.895
Creatinine, mg/dL	1.1 (0.8, 1.5)	1.0 (0.8, 1.4)	1.4 (1.0, 2.1)	<0.001
Prothrombin time, s	14.4 (12.6, 16.9)	14.4 (12.6, 16.8)	14.5 (12.9, 18.8)	0.002
Lactate, mg/dL	2.1 (1.4, 3.1)	2.1 (1.4, 3.0)	2.6 (1.5, 4.1)	<0.001
HbA1c, %	5.9 (5.5, 6.9)	5.9 (5.5, 6.9)	5.9 (5.5, 6.6)	0.068
TWAG, mg/dL	130 (115, 155)	129 (114, 154)	145 (131, 178)	<0.001
Glycemic distance, mg/dL	3.2 (-15.1, 20.1)	2.3 (-15.7, 18.8)	23.6 (3.33, 43.4)	<0.001
Diabetes, no (%)	2090 (44.1)	1958 (44.1)	132 (43.7)	0.929
Sepsis, no (%)	2625 (55.4)	2366 (53.3)	259 (85.8)	<0.001
Hypoglycemia, no (%)	522 (11.0)	464 (10.5)	58 (19.2)	<0.001
severe hypoglycemia, no (%)	25 (0.5)	16 (0.4)	9 (3.0)	<0.001
Treatment				
MV, no (%)	3157 (66.6)	2894 (65.3)	263 (87.1)	<0.001
MV duration, h	7.7 (0, 27.4)	6.9 (0, 23.0)	75.3 (29.4, 159)	<0.001
Vasopressors, no (%)	2484 (52.4)	2252 (50.8)	232 (76.8)	<0.001
RRT, no (%)	308 (6.5)	242 (5.5)	66 (21.9)	<0.001
Mean insulin dose, IU/day	9.1 (0, 32.2)	9.6 (0, 32.5)	4.8 (0.32, 24.4)	0.292
Length of ICU stay, days	3.9 (2.7, 6.1)	3.8 (2.7, 5.9)	6.2 (3.9, 10.7)	<0.001

Continuous variables are presented as median with interquartile range (IQR) and were compared between groups using Kruskal-Wallis tests. Categorical variables are presented as numbers and percentage and were compared between groups using the chi-square. Statistical significance was set at $p<0.05$. BMI, body mass index; MAP, mean arterial pressure; CCI, Charlson Comorbidity Index; APS III, Acute Physiology Score III; SOFA, Sequential Organ Failure Assessment; WBC, white blood cell; HbA1c, glycated hemoglobin; TWAG, time-weighted average glucose; MV, mechanical ventilation; RRT, renal replacement therapy.
